# Parental consanguinity and ovarian reserve: A retrospective cohort study

**DOI:** 10.18502/ijrm.v21i12.15039

**Published:** 2024-01-25

**Authors:** Shadya Al Saeghi, Tamadhir Al-Mahrouqi, Maha AL-Khadhuri, Rahma AL-Ghabshi, Jokha AL-Sabti, Sachin Jose, Vaidyanathan Gowri

**Affiliations:** ^1^Obstetrics and Gynecology Residency Training Program, Oman Medical Specialty Board, Muscat, Oman.; ^2^General Foundation Program, Oman Medical Specialty Board, Muscat, Oman.; ^3^Obstetrics and Gynecology Department, Sultan Qaboos University and Hospital, Muscat, Oman.; ^4^Obstetrics and Gynecology Department, Royal Hospital, Muscat, Oman.; ^5^Department of Research, Oman Medical Specialty Board, Muscat, Oman.

**Keywords:** Consanguinity, Ovarian reserves, Infertility, Female.

## Abstract

**Background:**

Infertility affects around 10-15% of couples worldwide and is both a social and medical problem. Parental consanguinity is considered to reduce fertility reserve. Consanguineous marriages, especially first cousin marriages, are very common in Oman according to the Oman National Health Survey data.

**Objective:**

This study aimed to determine whether women born to consanguineous parents have reduced ovarian reserve.

**Materials and Methods:**

This cohort study was conducted on 414 women aged ≤ 39, treated for infertility at Sultan Qaboos University hospital and Royal hospital, Muscat, Oman from January 2019-December 2020. Each participant was interviewed and a complete history, including parental consanguinity and physical examination, were recorded. On day 2 of the menstrual cycle, serum concentration of the following was performed: follicle-stimulating hormone (FSH), luteinizing hormone, estradiol, prolactin, thyroid stimulating hormone, and anti-Müllerian hormone (AMH). AMH was done, if necessary, on other days of the cycle. Antral follicle count (AFC) was done on day 2 and 3 of the menstrual cycle.

**Results:**

Of the 414 women, parental consanguinity was present in 40.2% of couples. In women with low AFC, parental consanguinity was present in 15.3% compared to 13.0% in the non-consanguineous group. About 15% of women with low AMH had consanguineous parents, compared to 20.2% from the non-consanguineous group. High levels of FSH were present in 6.5% and 4.2% of the consanguineous and non-consanguineous groups, respectively. No significant difference was observed in AFC with reference to body mass index.

**Conclusion:**

The results from this study showed no statistically significant difference in low ovarian reserves (AFC, AMH, and FSH) in women whose parents had a consanguineous marriage.

## 1. Introduction

Infertility is the inability to get pregnant even after a year of regular unprotected sexual intercourse. It affects around 10% of couples worldwide (1). Infertility is globally acknowledged as a health problem and has a social stigma attached to it. The Centers for Disease Control and Prevention emphasizes that infertility has considerable public health consequences, including psychological distress, social stigmatization, economic strain, and marital instability (2, 3).

Consanguineous marriage is a union between individuals related by blood as first or second cousins or as distant relatives. Marriages between first and second cousins account for over 10% of marriages worldwide (4), and about 8-10% of children worldwide have parents who are related consanguineous (3). The highest consanguinity rates were reported among “Pakistan army personnel and isolated Egyptian Nubians (76% and 80.4%), respectively” (5). A high rate of consanguineous marriages, similar to Asian and African countries are found in Qatar (54%), Saudi Arabia (52%), United Arab Emirates (50.5%), Sana'a in Yemen (44.7%), and Kuwait, 42.1% (6). The Omani National Health Survey indicated that up to 52% of marriages were consanguineous, the first cousins on the father's side or the mother's side were either parallel patrilateral or cross-cousins. The father's sister's is cross-cousin type I, and the mother's sister's son type II cross-cousin. There is a belief that consanguineous marriages cause fertility reduction (7). Some data available indicates that children of consanguineous parents run a 10 times greater risk of congenital defects and autosomal recessive diseases (8).

Studies on the association between consanguinity and fertility in Pakistan and India had conflicting results. The mean fertility was lower among Pakistani women born to first-cousin unions. In contrast, the mean fertility levels in India were similar in first cousin and non-consanguineous marriages (9). Another study from Egypt, which included women from Upper Egypt governorates, mentions a decline in ovarian reserve and infertility rates among offspring of consanguineous marriages (10).

Ovarian reserve in women refers to the number of good-quality eggs that determines their fertility potential. There are several ways to estimate the ovarian reserve, such as cycle day 3 follicle-stimulating hormone (FSH) levels, anti-Müllerian hormone (AMH) levels, and determining the ovarian antral follicle count (AFC) by doing a transvaginal ultrasound on day 2 of the cycle. None of them have a very high predictive value, and fertility experts often use a combination of tests to better estimate the size of the remaining egg supply in addition to the age of the women (11). AMH levels appear to be a more sensitive marker of ovarian reserve, and FSH is specific. AFC is a reasonable alternative in an experienced center (12).

According to a study in Kuwait in 2015, consanguinity and history of any surgery in non-consanguineous women were strong positive predictors of low ovarian reserve. They concluded that consanguinity in parents is strongly associated with reduced ovarian reserve (13).

This study aimed to investigate the impact of consanguineous marriage on women's fertility (ovarian reserve) in married couples. This study aimed to assess the association between parental consanguinity and ovarian reserve.

## 2. Materials and Methods

### Study design and participant selection

This multicenter retrospective cohort study was conducted in Muscat, Oman. The study included all women who were ≤ 39 yr, treated for infertility at the Sultan Qaboos University hospital, Muscat, Oman from January 2019-December 2020. Women who had received previous treatment that might have affected fertility (e.g., chemotherapy, radiotherapy, surgery on ovaries), women diagnosed with sickle cell disease or thalassemia, previous history of multiple blood transfusions or iron chelation agents, diagnosis of endometriosis, and those with premature ovarian failure were excluded.

### Sample size

The sample size was estimated based on the primary objective, the prevalence of history of consanguinity among infertile women who were undergoing treatment in 2 infertility clinics in Muscat. Therefore, the sample size was estimated based on the anticipated prevalence of 50% with an error of 5% and a confidence level of 95%. The sample size was estimated to be 377 using the online tool OpenEpi (https://www.openepi.com/SampleSize/SSPropor.htm). However, we have included 414 subjects.

### Data collection

Data were collected through an interview. Women's complete medical history, including age, medical history, surgical history, family history, and information on parental consanguinity and consanguinity in the couple themselves. Medical records were reviewed to collect the hormonal profile data, including FSH, luteinizing hormone, estradiol, prolactin, thyroid-stimulating hormone, and AMH serum concentration if available. A routine ultrasound assessment of the pelvis was performed on day 2 of the cycle to exclude any obvious pelvic pathology. The ovaries were visualized in the longitudinal plane, and the antral follicles measuring 2-10 mm in diameter within each ovary was counted separately to calculate the AFC. A single observer with the same machine did all the ultrasound scans for AFC at each site. AFC was done on cycle day 2 or 3 as far as possible. Serum luteinizing hormone and FSH (IU/l) were measured using the automated Elecsys immune analyzer, and AMH concentration levels were determined using the enzyme immunometric assay. The low ovarian reserve was defined as FSH of ≥ 10 IU/l, AMH of < 5 pmol, and AFC ≤ 7.

### Ethical considerations

The study was approved by the Ethics and Research Committee of Sultan Qaboos University, Muscat, Oman (MERC1806) and by the Ethics and Research Committee of the Royal hospital, Muscat, Oman (SRC84/2020). All participants signed an informed consent form.

### Statistical analysis

The categorical variables were presented as frequency and percentage. The association between categorical variables were assessed using chi-square test (Fisher's exact/Likelihood ratio). A multivariate binary logistic regression analysis was performed to determine the independent predictors of low ovarian reserve. A box plot was used for the graphical presentation of the association between consanguinity and AFC. P < 0.05 was considered statistically significant. All the analysis was carried out in IBM SPSS Statistics (IBM Corp. Released 2021. IBM SPSS Statistics for Windows, Version 28.0. Armonk, NY: IBM Corp) version 28.0.

## 3. Results

**Figure 1:**
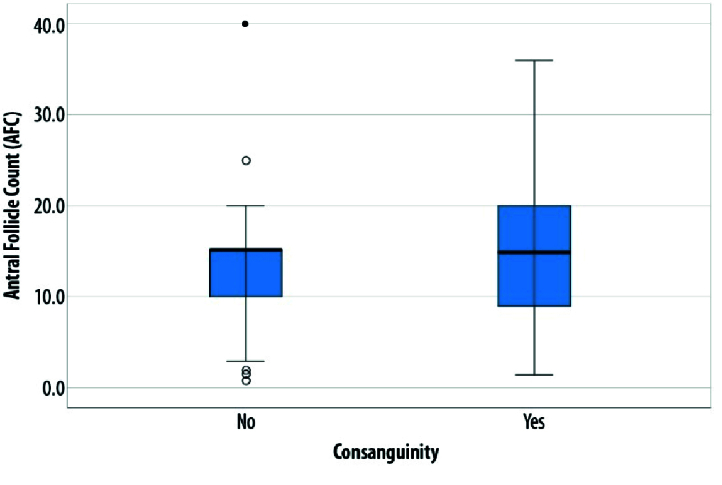
Relationship between consanguinity and AFC.

The total sample size was 414 from 2 hospitals. In this study, 40.2% of the couples had a history of consanguinity among their parents. About 38% of couples themselves were consanguineous. Around 23.5% of participants had medical comorbidities (Table I). A significant age difference was observed between women with low AFC and those with normal AFC. About 15.0% of women with a history of parental consanguinity had low AFC, compared to around 13% of participants with no parental history of consanguinity, which was not statistically significant (Table II). Unfortunately, due to lack of funding, it was possible to estimate AMH only in 40% of the sample (181 of 414). Around 15% of women with low AMH had a history of consanguineous parents compared to 20.2% in the non-consanguineous group. FSH levels were estimated in almost 90% of the sample, and AFC was done in about 71% of women. High FSH level was found in 6.7% of the consanguineous group and 4.1% of the non-consanguineous group. No statistically significant difference was observed in the women concerning low ovarian reserve and parental consanguineous marriage (Table III). The multivariate regression model had failed to identify any significant predictors for the low ovarian reserve, although age and FSH were statistically significant in the univariate analysis (Table IV). The box plot depicts the relationship between consanguinity and AFC (Figure 1).

**Table 1:** Characteristics of the infertile women and parental consanguinity


**Variables**		**History of parental consanguinity**		
		**No (n = 244)**	**Yes (n = 170)**	**P-value**
**Age (yr)**				
	**20-30**	124 (50.8)	78 (45.9)	
	**> 30**	120 (49.2)	92 (54.1)	0.368
**BMI (kg/m^2^)**				
	**< 18.50**	6 (2.5)	6 (3.6)	
	**18.50-24.99**	73 (30.5)	45 (26.9)	
	**25.00-29.99**	62 (25.9)	46 (27.5)	
	**≥ 30.00**	98 (41.1)	70 (42.0)	0.816
**Years of infertility (yr)**				
	**< 4**	95 (38.9)	67 (39.4)		
	**5-10**	101 (41.4)	71 (41.8)		
	**> 10**	48 (19.7)	32 (18.8)	0.977
**Type of infertility**				
	**Primary**	142 (58.2)	91 (53.5)		
	**Secondary**	102 (41.8)	79 (46.5)	0.366
Data presented as n (%). Chi-square test. BMI: Body mass index				

**Table 2:** Association between low AFC and the participants' characteristics


**Variable**		**Low ovarian reserve**		
		**No ( > 7), n = 254**	**Yes ( ≤ 7), n = 41**	**P-value**
**Age (yr)**				
	**20-30**	141 (55.5)	13 (31.7)	
	**> 30**	113 (44.5)	28 (68.3)	< 0.001*
**BMI (kg/m^2^)**				
	**< 18.50**	8 (3.2)	1 (2.4)	
	**18.50-24.99**	56 (22.5)	17 (41.5)	
	**25.00-29.99**	66 (26.5)	9 (22.0)	
	**≥ 30.00**	119 (47.8)	14 (34.1)	0.100
**Years of infertility (yr)**				
	**< 4**	104 (40.9)	13 (31.7)		
	**5-10**	107 (42.2)	19 (46.3)		
	**> 10**	43 (16.9)	9 (22.0)	0.490
**Type of infertility**				
	**Primary**	151 (59.4)	18 (43.9)		
	**Secondary**	103 (40.6)	23 (56.1)	0.088
**History of consanguinity**				
	**No**	157 (61.8)	24 (58.5)	
	**Yes**	97 (38.2)	17 (41.5)	0.731
**FSH**				
	**≤ 10 (normal)**	226 (97.8)	34 (89.5)	
	**> 10 (low)**	5 (2.2)	4 (10.5)	0.025*
Data presented as n (%). Chi-square test, *Statistically significant. AFC: Antral follicle count, BMI: Body mass index, FSH: Follicle-stimulating hormone				

**Table 3:** Association between ovarian reserve levels and the history of parental consanguinity


**Variable**		**History of parental consanguinity**		
		**Yes**	**No**	**P-value**
**AMH**				
	**< 5 (low)**	12 (15.4)	16 (18.2)	
	**≥ 5 (normal)**	66 (84.6)	72 (81.8)	0.682
**FSH**				
	**≤ 10 (normal)**	140 (93.3)	212 (95.9)	
	**> 10 (low)**	10 (6.7)	9 (4.1)	0.338
**AFC**				
	**Normal (> 7)**	97 (85.1)	157 (86.7)	
	**Low (≤ 7)**	17 (14.9)	24 (13.3)	0.731
Data presented as n (%). Fisher's exact test. AMH: Anti-Müllerian hormone, FSH: Follicle-stimulating hormone, AFC: Antral follicle count				

**Table 4:** Multivariate binary logistic regression analysis was performed to determine the independent predictors of low ovarian reserve


**Variable**			**95% CI for OR**	
		**β**	**P-value**	**OR**	**Lower**	**Upper**
**Age** **(yr)**						
	**20-30 (Reference)**			
	**> 30 **	0.690	0.116	1.994	0.844	4.713
**BMI (kg/m^2^**)						
	**< 18.50**	0.218	0.846	1.244	0.138	11.192
	**18.50-24.99**	0.846	0.056	2.331	0.977	5.561
	**25.00-29.99**	0.090	0.854	1.094	0.420	2.847
	**≥ 30.00 (Reference)**			
**Years of infertility**						
	**< 4 yr (Reference)**			
	**5-10 yr**	0.179	0.692	1.196	0.493	2.900
	**> 10 yr**	0.111	0.854	1.118	0.342	3.652
**History of consanguinity**						
	**No (Reference)**			
	**Yes**	0.552	0.182	1.736	0.773	3.900
**FSH**						
	**≤ 10 (normal) (Reference)**			
	**> 10 (low)**	1.323	0.081	3.756	0.849	16.627
Nagelkerke R^2^ = 0.114, overall accuracy = 86.8%. BMI: Body mass index, FSH: Follicle-stimulating hormone, OR: Odds ratio						

## 4. Discussion

In this study, about 40% of infertile couples had parents with consanguinity. However, none of the markers of ovarian reserve like AFC or AMH had significant differences between the 2 groups, consanguineous and non-consanguineous. Women whose parents were first- or second-degree cousins were classified as parental consanguinity, and non-consanguinity was defined as parents who were not related for at least 3 generations.

Results from this study showed that the prevalence of consanguineous marriages in the Omani community is similar to previous reports from the Omani National Health Survey in 2000 (5) and other Gulf countries (4). This tradition appears common with a strong desire for consanguineous marriages. This practice is maintained to help family structure and property, and strengthen family ties; in some families, they see it as an advantage relating to the bridal dowry. This is seen as a way of reducing marital conflicts and helping to maintain stability and durability in relationships (6).

In this study, various recognized parameters of ovarian reserve assessments were used, as no single test is accurate enough for ovarian reserve assessment. The important role of AMH estimation as one of the most reliable biomarkers for ovarian reserve assessment has previously been noted, with AMH levels markedly declining with advancing age (14). However, in addition to AMH, other tests like FSH and AFC were carried out, as recommended by many previous studies. Our results showed no significant difference in ovarian reserve parameters between the parental consanguineous and non-consanguineous groups. Conversely, studies from Kuwait (13) and Egypt (10) showed reduced ovarian reserves in women born of consanguineous marriages.

Testing the AFC is one of the most accurate non-invasive measures to test ovarian aging. Previous studies have demonstrated that an AFC < 7 is a risk factor for infertility (11, 12). Therefore, in this study, participants were classified into normal AFC ( > 7) and reduced AFC ( ≤ 7) categories. In this study, 84% of the consanguineous parents group and 87% of the non-consanguineous group had an AFC > 7, this was not statistically significant. In a study from Kuwait, 29.9% of participants with consanguineous parents had an AFC ≥ 9, compared with 63.9% in the non-consanguineous group (13).

Clinical studies have demonstrated that serum AMH levels correlate strongly to AFC and are more accurate than age and other hormonal measurements like FSH, estradiol, and Inhibin in predicting the success of ovulation induction, especially in a set-up with assisted reproduction. Despite controversies in the assay of AMH levels, the decline in AMH levels is interpreted as the first indication of a decline in the ovarian follicular reserve. Notably, AMH concentration remains stable throughout the menstrual cycle (15). In this study, around 15% of women with low AMH had a history of consanguineous parents compared to 20.2% of the non-consanguineous group, which was not statistically significant. However, due to financial constraints, AMH levels were not tested in all participants, as it is not routinely done in the Omani health system due to financial constraints. This is one limitation of this study, though FSH and AFC were done in most women.

High FSH levels were noted in 6.7% of the consanguineous parent group compared to 4.1% in the non-consanguineous group, which was expected, as FSH is known to be a late marker of ovarian aging and follows a monthly variation pattern (13).

## 5. Conclusion

In conclusion, our study indicated that parental consanguinity does not affect their female offspring's ovarian reserve.

##  Conflict of Interest 

The authors declare that there is no conflict of interest.

## References

[bib1] Akhondi MM, Ranjbar F, Shirzad M, Behjati Ardakani Z, Kamali K, Mohammad K (2019). Practical difficulties in estimating the prevalence of primary infertility in Iran. Int J Fertil Steril.

[bib2] Sun H, Gong T-T, Jiang Y-T, Zhang Sh, Zhao Y-H, Wu Q-J (2019). Global, regional, and national prevalence and disability-adjusted life-years for infertility in 195 countries and territories, 1990-2017: Results from a Global Burden of Disease Study, 2017. Aging (Albany NY).

[bib3] Zhang L, Shao H, Huo M, Chen J, Tao M, Liu Zh (2022). Prevalence and associated risk factors for anxiety and depression in infertile couples of ART treatment: A cross-sectional study. BMC Psychiatry.

[bib4] Mazharul Islam M (2017). Consanguineous marriage in Oman: Understanding the community awareness about congenital effects of and attitude towards consanguineous marriage. Ann Hum Biol.

[bib5] Iqbal S, Zakar R, Fischer F, Zakar MZ (2022). Consanguineous marriages and their association with women’s reproductive health and fertility behavior in Pakistan: Secondary data analysis from demographic and health surveys, 1990-2018. BMC Women's Health.

[bib6] Al-Ghanim KA (2020). Consanguineous marriage in the Arab societies. J Psychol Clin Psychiatry.

[bib7] Romdhane L, Mezzi N, Hamdi Y, El-Kamah G, Barakat A, Abdelhak S (2019). Consanguinity and inbreeding in health and disease in North African Populations. Annu Rev Genom Hum Genet.

[bib8] Anwar S, Taslem Mourosi J, Arafat Y, Hosen MJ (2020). Genetic and reproductive consequences of consanguineous marriage in Bangladesh. PLoS One.

[bib9] Jaber L, Nashif AS, Diamond G (2023). Consanguinity, fertility and reproductive outcomes: An international review. Med Res Arch.

[bib10] Hussein WM, El-Gaafary MM, Wassif GO, Wahdan MM, Sos DG, Hakim SA, et al (2022). Correlates and reproductive consequences of consanguinity in six Egyptian governorates. Afr J Reprod Health.

[bib11] Findlay JK, Hutt KJ, Hickey M, Anderson RA

[bib12] Practice Committee of the American Society for Reproductive Medicine (2020). Testing and interpreting measures of ovarian reserve: A committee opinion. Fertil Steril.

[bib13] Seher T, Thiering E, Al Azemi M, Heinrich J, Schmidt-Weber CB, Kivlahan C, et al

[bib14] Iwase A, Nakamura T, Osuka S, Takikawa S, Goto M, Kikkawa F

[bib15] Moolhuijsen LME, Visser JA (2020). Anti-Müllerian hormone and ovarian reserve: Update on assessing ovarian function. J Clin Endocrinol Metab.

